# LIN28B promotes the progression of endometrial cancer through upregulating MYC and correlates with immune microenvironment

**DOI:** 10.3389/fonc.2025.1592193

**Published:** 2025-07-16

**Authors:** Yuchao Diao, Xiangkun Li, Chang Wang, Jinwen Jiao, Dongmei Gao, Weifang Mao, Xinping Yu, Hongjuan Yang

**Affiliations:** ^1^ Department of Gynecology, The Affiliated Hospital of Qingdao University, Qingdao, China; ^2^ Department of Anesthesiology, The Affiliated Hospital of Qingdao University, Qingdao, China

**Keywords:** LIN-28 homolog B, endometrial cancer, MYC, prognosis, immune microenvironment

## Abstract

**Background:**

Endometrial cancer (EC), a leading gynecologic malignancy, demonstrates a rising global incidence that imposes significant clinical and socioeconomic burdens. While the RNA-binding protein LIN28B has been reported to promote the progression of EC, its mechanistic role in driving tumor progression and immune modulation remains poorly characterized. This study specifically investigates whether LIN28B promotes EC progression through MYC upregulation and its influence on tumor immune microenvironment remodeling.

**Methods:**

Utilizing integrated bioinformatics analysis of TCGA/GTEx datasets and immunohistochemical staining of clinical specimens, we evaluated LIN28B expression in EC. Survival outcomes associated with LIN28B were analyzed using the Kaplan-Meier methodology. Functional validation was conducted in HEC-1A, HEC-1B, and KLE cell lines through siRNA-mediated LIN28B knockdown. Proliferative capacity (CCK-8 and EdU assays), clonogenic potential (colony formation assay), and metastatic behavior (Transwell assays) were systematically assessed. Mechanistic studies employed quantitative real-time PCR and Western blotting to confirm LIN28B-mediated regulation of MYC, with further validation via rescue experiments combining LIN28B overexpression and MYC silencing. Immune microenvironment alterations linked to LIN28B expression were profiled using ssGSEA implemented via the GSVA package. Finally, a multivariate prognostic nomogram incorporating LIN28B expression and clinicopathological parameters was constructed and calibrated using Cox regression modeling and calibration curves.

**Results:**

LIN28B exhibited significant overexpression in EC tissues and was associated with diminished overall survival, progression-free survival, and disease-specific survival. Functional analyses revealed that LIN28B knockdown markedly suppressed EC cell proliferation, migration, and invasion, concurrent with MYC downregulation. MYC depletion abrogated LIN28B-driven oncogenic effects, validating their functional dependency. Immune profiling identified that elevated LIN28B expression correlated with reduced infiltration of thirteen distinct immune cell subsets. A multivariate prognostic nomogram combining LIN28B expression and clinicopathological parameters established a robust predictive model for EC outcomes.

**Conclusion:**

LIN28B exhibits oncogenic roles in EC by facilitating MYC-mediated tumor progression and modulating the immune microenvironment, establishing its potential as both a therapeutic target and a prognostic biomarker.

## 1 Introduction

Endometrial cancer (EC) is a prevalent malignancy, with 65,620 new cases and 12,590 deaths reported in the United States in 2020, while China and other regions demonstrate comparable epidemiological trends (1, 2). Although 80-85% of patients achieve favorable outcomes through early detection, approximately 15-20% develop aggressive metastatic disease with elevated mortality (3). These clinical realities underscore the urgent need to elucidate EC pathogenesis and develop targeted interventions.

The RNA-binding protein Lin-28 homolog B (LIN28B), first characterized in hepatocellular carcinoma, functions as an oncogenic driver through selective inhibition of let-7 tumor-suppressive microRNA maturation (4). This molecular blockade activates downstream oncogenic pathways (MYC, E2F, RAS), driving malignant progression in colorectal, glioblastoma, and ovarian malignancies (5, 6). LIN28B enhances therapeutic resistance and tumor recurrence by potentiating proliferation and epithelial-mesenchymal transition, positioning it as both a predictive biomarker and therapeutic target (7). Recent studies have demonstrated that LIN28B promotes the progression of EC (8). However, the underlying molecular mechanisms of LIN28B-driven EC pathogenesis and its potential immunomodulatory functions within the tumor microenvironment remain poorly characterized.

Emerging evidence reveals a self-reinforcing LIN28B-MYC regulatory axis: MYC transcriptionally activates LIN28B expression, while LIN28B post-transcriptionally stabilizes MYC by inhibiting the maturation of let-7 miRNA (9–12). Despite this well-characterized oncogenic circuit in other malignancies, its functional significance in EC remains unexplored.

To address these unsolved questions, this study systematically investigates the mechanistic basis of LIN28B-driven tumorigenesis in EC, focusing on its MYC-dependent oncogenic axis and immunomodulatory roles. Using integrated bioinformatics analysis of TCGA/GTEx datasets and functional validation in EC cell models, we investigate the molecular interplay between LIN28B and MYC and evaluate its impact on tumor immune microenvironment reconfiguration. Additionally, we develop and validate a multivariate prognostic model integrating LIN28B expression with established clinicopathological parameters. This study aims to advance the characterization of LIN28B in EC pathogenesis, systematically investigating its dual potential as both a therapeutic vulnerability and a predictor of clinical outcomes.

## 2 Materials and methods

### 2.1 Cell culture

The EC cell lines (HEC-1A, HEC-1B, and KLE) were obtained from the American Type Culture Collection and cultured in RPMI-1640 medium (Invitrogen), containing 10% FBS (Invitrogen). The cells were incubated in a bioreactor environment maintained at 37°C with 5% carbon dioxide.

### 2.2 RNA interference and lentiviral infection

LIN28B-specific small interfering RNAs (siRNA) were acquired from the Shanghai Shenggong Biological Organization. The transfection of cells with these siRNAs was conducted utilizing Lipofectamine 3000 reagent (Invitrogen). Additionally, LIN28B expression vectors were obtained from VectorBuilder, while lentiviral LIN28B shRNA plasmids were sourced from Sigma. For a period of 24 hours, KLE cells were subjected to lentiviral infection. Subsequently, they were selected with puromycin (2µg/ml) for a duration of 5 to 7 days.

### 2.3 Quantitative real-time PCR

RNA extraction was performed employing TRIzol reagent (Invitrogen), followed by the synthesis of cDNA using PrimeScript RT Master Mix (Takara). Subsequently, qRT-PCR was conducted utilizing SYBR Green qPCR master mix (Takara). The primer sequences utilized are detailed in **Supplementary Table S1**.

### 2.4 Western blotting

Protein extraction was performed on the cells utilizing RIPA solution (Beyotime), followed by quantification of the protein concentration with a BCA kit (Beyotime). Briefly, SDS-PAGE was employed to separate the proteins, which were then transferred to a PVDF membrane. Subsequently, the membrane underwent a blocking procedure with skim milk for 1.5 hours. The primary antibodies were incubated overnight and then probed with secondary antibodies for 2 hours. The primary antibodies utilized are detailed in **Supplementary Table S2**.

### 2.5 Cell Counting Kit-8 assay

We utilized the CCK-8 assay to assess the viability of EC cells. Cells were transferred to a 96-well plate. Subsequently, each well received 10 μL CCK-8 solution and incubated for 2.5 hours at standard temperature conditions. The absorbance reading was obtained at 450 nm.

### 2.6 Colony Formation assay

Cells were seeded at a density of 500 cells per well in 6-well plates. Following a 14-day incubation, cells underwent fixation in methanol for a duration of 20 minutes and were stained using crystal violet (Beyotime). The clonogenic potential was evaluated by counting and analyzing the number of cell colonies.

### 2.7 EdU assay

An EdU proliferation assay was conducted using the EdU Kit (Beyotime). 15,000 cells were seeded into each well of 96-well plate. Subsequently, a 1:1000 dilution of EdU reagent was added. After an incubation period of two hours, the cells underwent fixation with 4% paraformaldehyde and were stained with fluorescent dye and Hoechst.

### 2.8 Migration and invasion assay

The migration and invasion abilities were assessed using transwell assay. 1×105 cells were plated in the upper compartment, which contained serum-free media. The lower compartment was filled with medium supplemented with 20% FBS. Following incubation for 6–48 hours, cells were subjected to fixation with methanol and staining with crystal violet. The number of invaded and migrated cells was then quantified.

### 2.9 Immunohistochemical staining assay

The paraffin-embedded specimens of EC tumor tissues and adjacent non-tumor tissues (*n*=8) used in this study were obtained from The Affiliated Hospital of Qingdao University. The study protocol was approved by the Ethics Committee of The Affiliated Hospital of Qingdao University (Approval No. QYFY-WZLL-29957) and conducted in compliance with the Declaration of Helsinki. For IHC analysis, 4-μm-thick sections underwent antigen retrieval in citrate buffer (pH 6.0) using a pressurized decloaking chamber for 5 minutes. Endogenous peroxidase activity was quenched with 3% hydrogen peroxide incubation for 10 minutes. After blocking with 10% goat serum at room temperature for 30 minutes, sections were incubated with primary antibodies (anti-LIN28B, Invitrogen, PA5-63983; anti-MYC, Abcam, ab32072) for 1 hour at room temperature. Then secondary antibodies were applied for 15 minutes. Nuclear counterstaining was subsequently performed using hematoxylin.

### 2.10 Bioinformatics analysis

The TCGA-TPM data analyzed in this study were derived from TCGA-UCEC STAR-Counts and preprocessed using the TOIL pipeline (https://portal.gdc.cancer.gov). TCGA-GTEx TPM data were sourced from the UCSC Xena platform (https://xenabrowser.net/datapages/) and similarly standardized through the TOIL pipeline (13). LIN28B expression levels were compared between tumor and normal samples using both unpaired (Wilcoxon rank sum test) and paired (Wilcoxon signed-rank test) non-parametric statistical methods. Patients were stratified into low- and high-LIN28B expression groups using the median expression value of LIN28B as the threshold. Subsequently, Kaplan-Meier survival analysis was conducted to determine the overall survival (OS), progression-free interval (PFI), and disease-specific survival (DSS). We utilized the DESeq2 package to identify differentially expressed genes (DEGs) in the low- versus high-LIN28B expression groups (14). Genes that satisfied both adjust p-value (padj) < 0.05 and |log fold change (FC) | > 1.5 were regarded as DEGs. GO and KEGG analyses were performed using the org.Hs.eg.db and ClusterProfiler packages for functional annotation, with the GOplot and ggplot2 packages employed for visualization (15, 16). Immune infiltration analysis was conducted using ssGSEA via the GSVA package, utilizing TCGA data (17). We performed the correlation analysis between LIN28B and immune cells using the Spearman correlation coefficient. Comparing groups with low and high levels of LIN28B expression using the Wilcoxon rank-sum test allowed us to determine which had a higher infiltration level of immunocytes. The Spearman correlation coefficient was utilized to determine the correlation analysis between LIN28B expression and immune cell infiltration (18). Cox regression analysis was utilized to assess the predictive ability of various variables on OS in EC and to identify independent prognostic factors. We detected independent prognostic variables, using multivariate Cox regression analysis with all significant parameters (*P*<0.1). Additionally, a nomogram prognostic model based on LIN28B expression was constructed through the application of the rms and survival packages. The model’s prediction accuracy was graphically assessed using calibration curves. The decision curve analysis (DCA) comparing models with and without LIN28B expression was performed with stdca.R package (19).

### 2.11 Statistical analysis

*In vitro* experimental data and clinical immunohistochemistry results were expressed as the mean ± standard deviation (SD) following confirmation of normality via the Shapiro–Wilk test. Variance homogeneity was verified using Levene’s test prior to ANOVA. For clinical samples with matched pairs, paired t-tests were employed, whereas one-way ANOVA with Bonferroni correction was applied to independent *in vitro* experimental groups. All statistical analyses were conducted using GraphPad Prism 9 (GraphPad Software, USA), with significance thresholds set as *P* < 0.05.

## 3 Results

### 3.1 LIN28B overexpression correlates with adverse prognosis in EC

**Figure 1:**
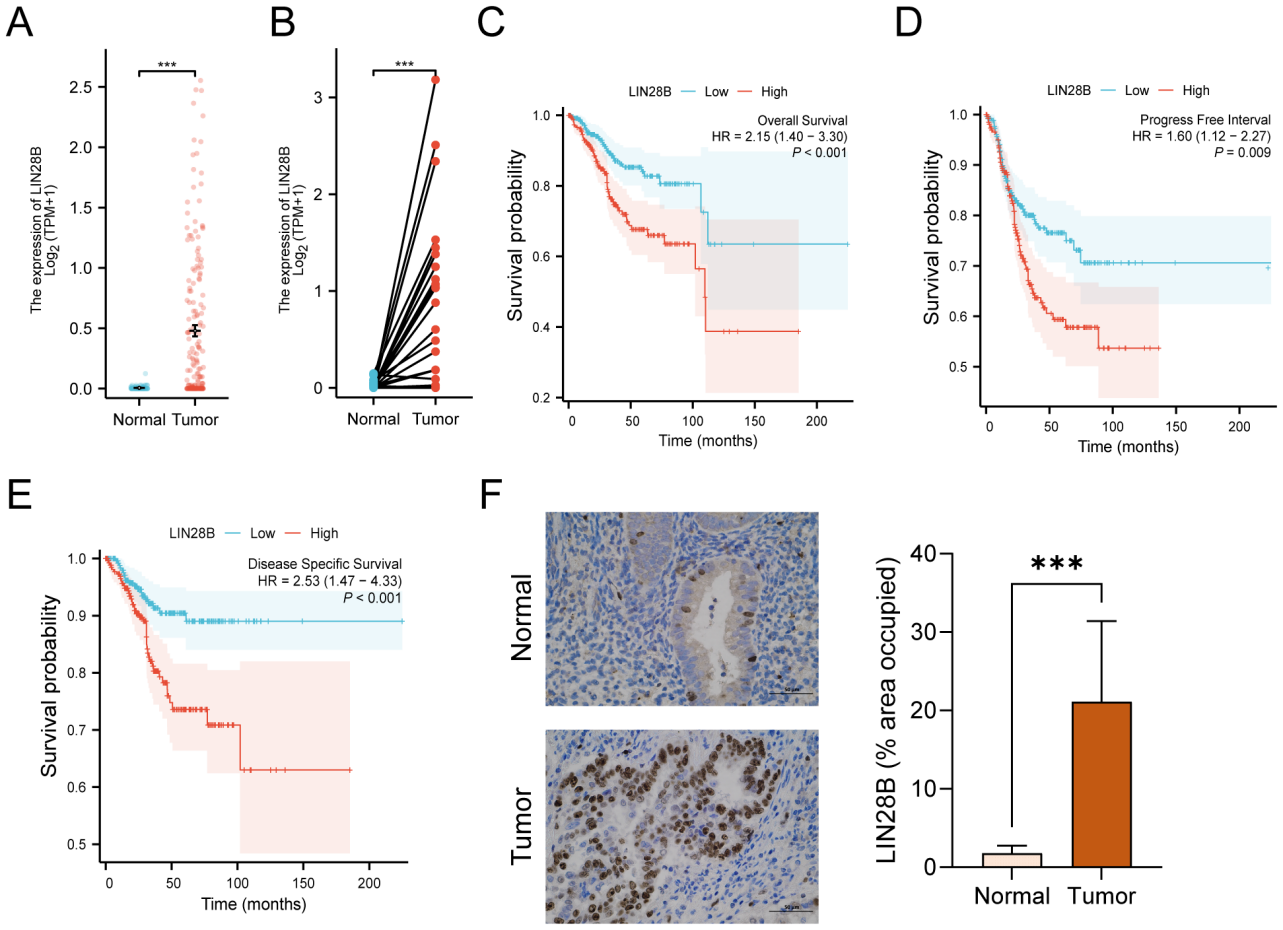
LIN28B overexpression correlates with adverse prognosis in EC. **(A)** Comparative analysis of LIN28B mRNA expression in normal samples of TCGA combined with GTEx versus EC samples of TCGA. **(B)** Differential LIN28B expression in paired tumor-normal specimens from EC patients of TCGA. **(C–E)** KM curves of OS, DSS, and PFI for EC patients. **(F)** IHC analysis of LIN28B in normal and EC tissues (×400, scale bar: 50 μm). The data are presented as the mean ± SD and assessed by conducting paired t-test, *n =* 8 for each group; ***P < 0.001.

A recent study has demonstrated that LIN28B expression in EC is significantly elevated compared to normal tissues through analyses of the TCGA database, with elevated LIN28B levels correlating with poorer OS (8). In this study, we further compared mRNA expression profiles from TCGA and GTEx databases and performed comprehensive analyses of PFI and DSS in patients with high LIN28B expression. As depicted in **Figure 1A**, the expression levels of LIN28B were markedly elevated in EC tissues (*n*=181) versus normal tissues (*n*=101). Analysis of LIN28B expression in paired tumor and normal tissues (*n*=23) from the TCGA database revealed a significant upregulation of LIN28B expression in tumor tissues (**Figure 1B**). Additionally, IHC analysis of eight paired tumor and adjacent normal tissues revealed markedly elevated LIN28B expression in tumor tissues (**Figure 1F**). The KM method was used to investigate the correlation between the expression level of LIN28B and EC prognosis. Consistent with prior findings, elevated LIN28B expression was significantly associated with poorer OS (**Figure 1C**). Our analysis extends these observations, establishing that high LIN28B expression predicts reduced PFI, and DSS (**Figures 1D–F**). Our results suggest that the increased LIN28B expression correlates with a poor prognosis of EC.

### 3.2 LIN28B knockdown suppresses EC cell proliferation

**Figure 2:**
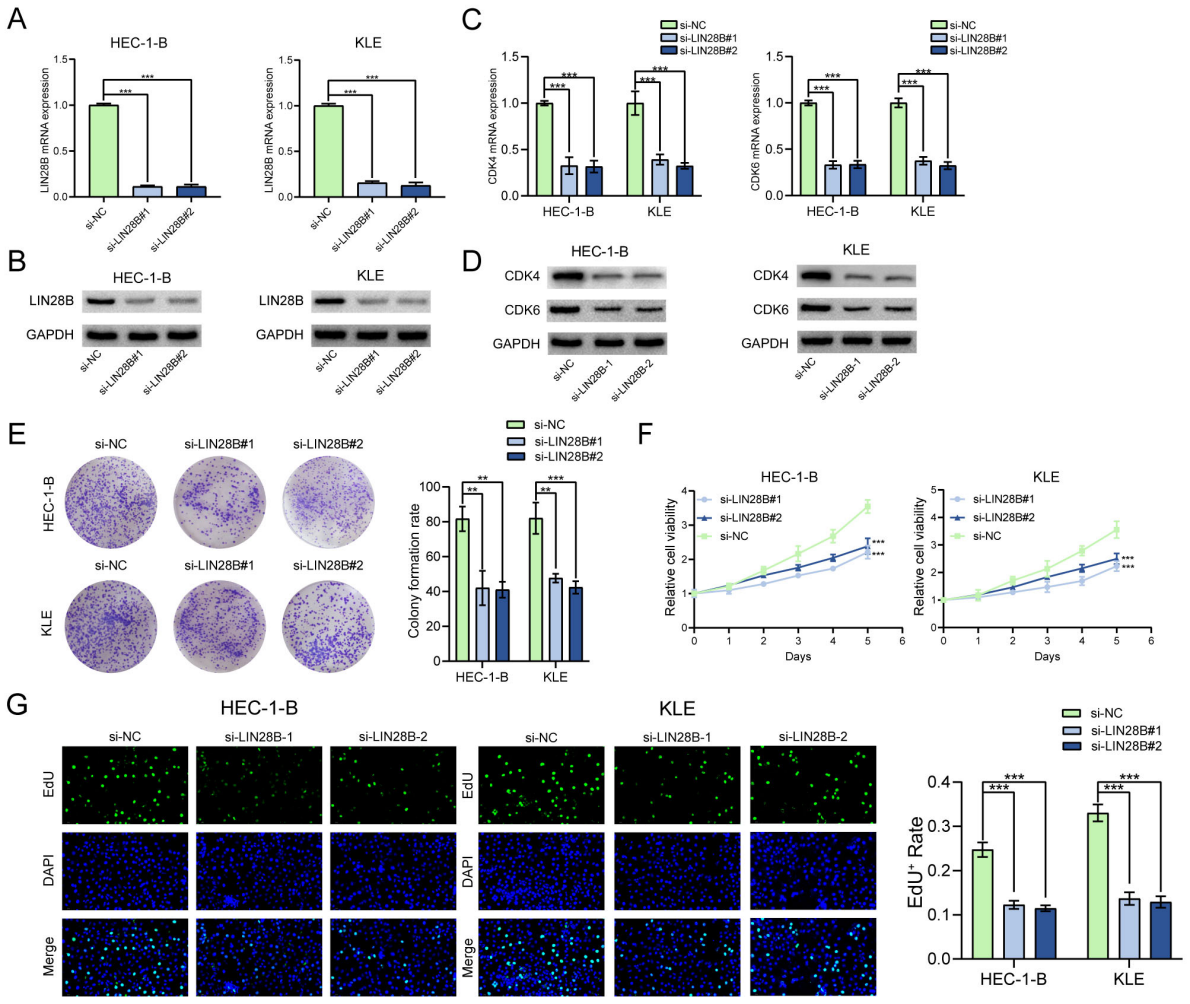
LIN28B knockdown suppresses EC cell proliferation. **(A, B)** Validation of LIN28B knockdown efficiency through qRT-PCR and WB assays. **(C, D)** CDK4 and CDK6 expression levels were evaluated by qRT-PCR and WB assays. **(E)** A Clone formation assay was utilized to assess the effect of LIN28B on clonality in EC cells. **(F)** CCK-8 assay was conducted to evaluate the impact of LIN28B on the viability of EC cells. **(G)** EdU assay was performed to assess the proliferation of EC cells. The data are presented as the mean ± SD and assessed by conducting one-way ANOVA and the Bonferroni correction for multiple comparisons, *n =* 3 for each group; ***P < 0.001.

To further elucidate the biological effect of LIN28B on EC, we constructed cell lines with reduced expression of LIN28B. Stable LIN28B knockdown models were validated via qRT-PCR and WB assays (**Figures 2A, B**) and these cell lines were utilized in subsequent experiments to validate the functional role of LIN28B in EC. Silencing LIN28B downregulated CDK4/6 mRNA and protein levels (**Figures 2C, D**), concomitant with reduced colony formation capacity in EC cells (**Figure 2E**). Additionally, CCK-8 and EdU assays revealed that silencing LIN28B significantly decreased the rate of cell growth, indicating a positive role for LIN28B in EC cell proliferation (**Figures 2F, G**).

### 3.3 LIN28B knockdown impairs EC cell migration and invasion.

**Figure 3:**
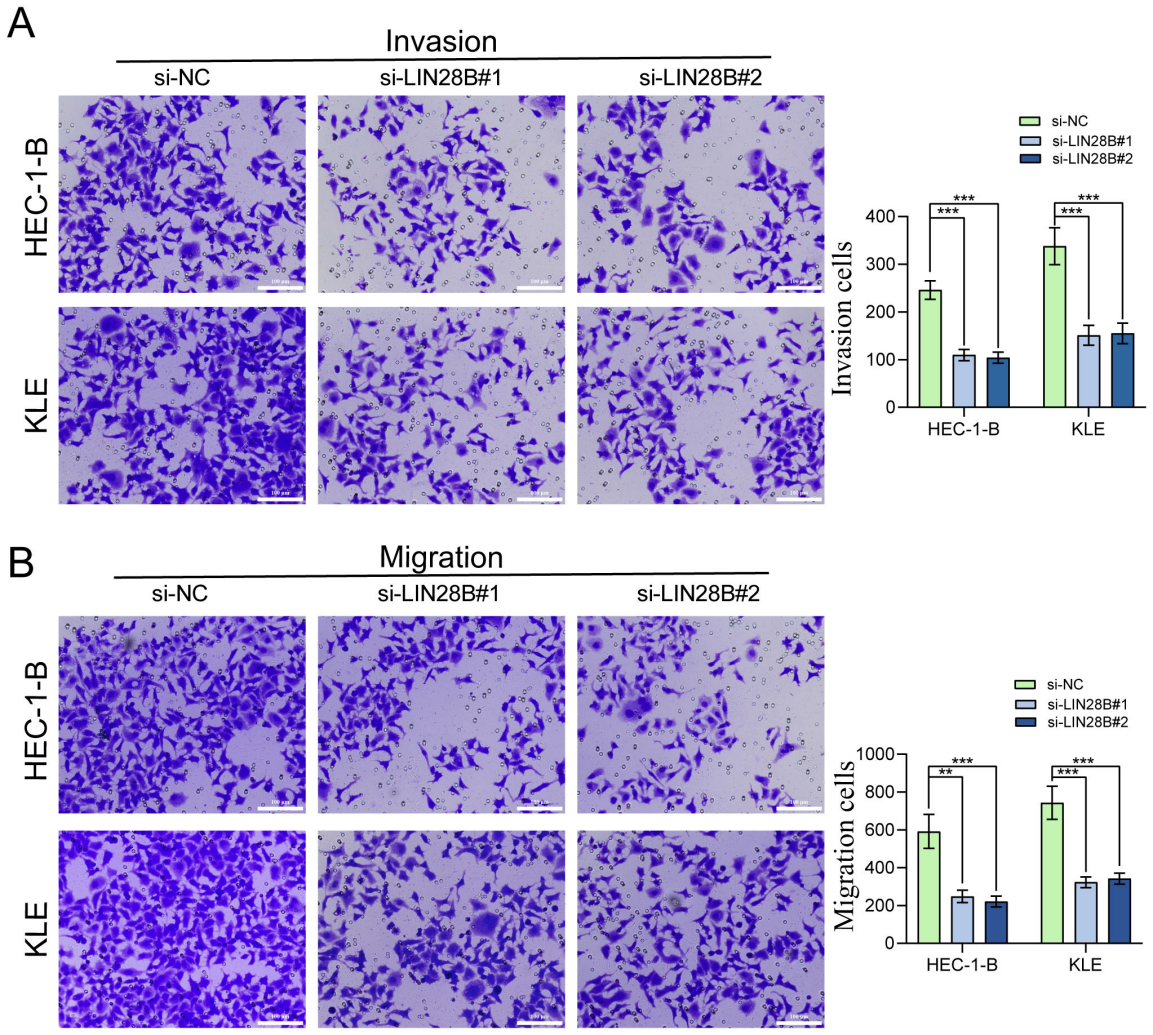
LIN28B knockdown impairs EC cells migration and invasion. **(A)** Evaluation of LIN28B-dependent invasive capacity was conducted using Transwell assay. **(B)** Evaluation of LIN28B-dependent migration ability was conducted using Transwell assay. The data are presented as the mean ± SD and assessed by conducting one-way ANOVA and the Bonferroni correction for multiple comparisons, *n =* 3 for each group; **P < 0.01, and ***P < 0.001.

We performed transwell assay to investigate the migration and invasion capabilities of EC cells. The results showed a great reduction in both migrating and invading cells after LIN28B silencing, indicating impaired migratory and invasive potentials (**Figures 3A, B**).

### 3.4 MYC deficiency impairs the biological function of LIN28B overexpression

**Figure 4:**
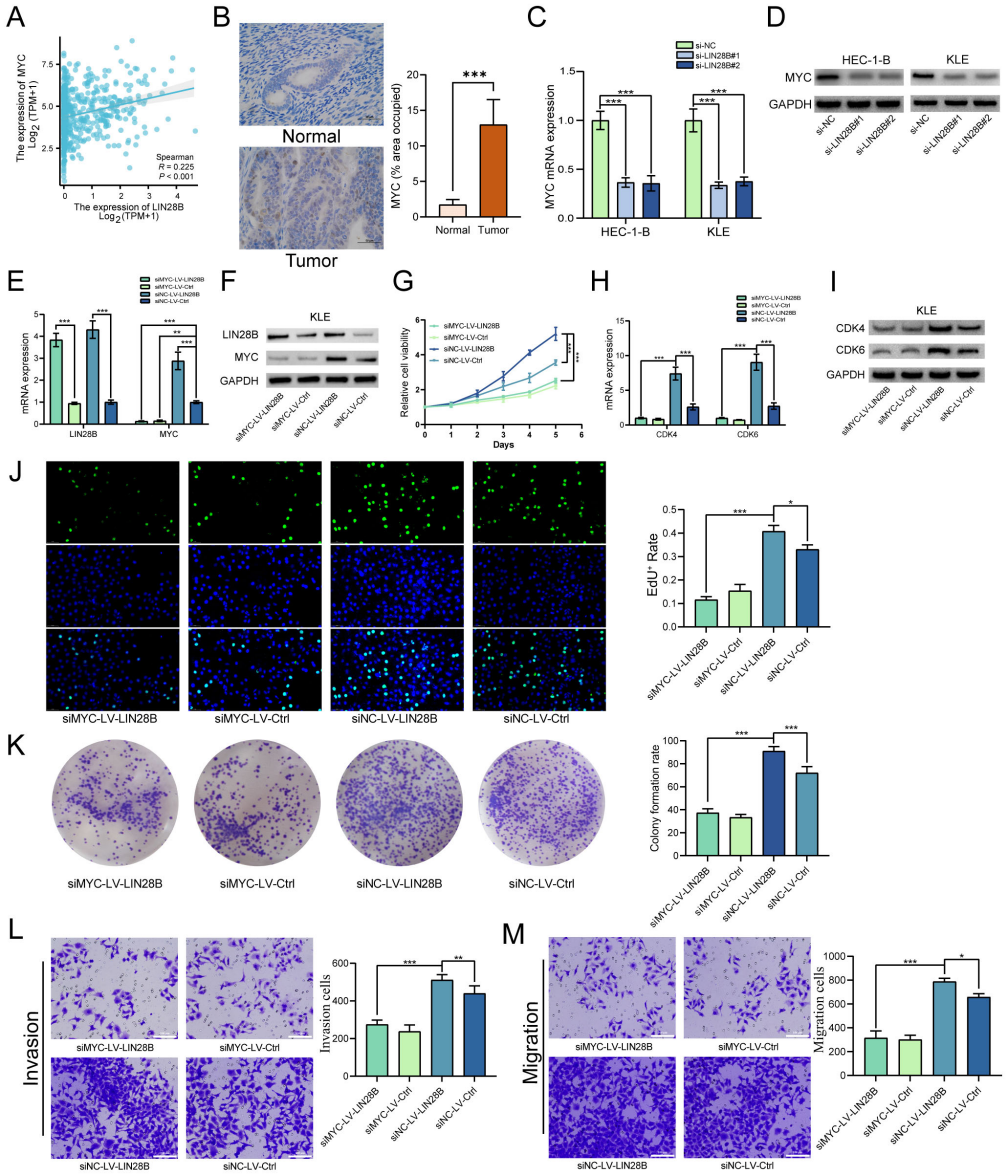
MYC deficiency impairs the biological function of LIN28B overexpression. **(A)** Correlation analysis of LIN28B and MYC mRNA expression in EC tissues from TCGA database. **(B)** IHC analysis of MYC in normal and EC tissues (×400, scale bar: 50 μm) (*n*=8). **(C, D)** LIN28B upregulates MYC expression as demonstrated by qRT-PCR and WB analyses. **(E, F)** LIN28B and MYC expressions were evaluated by qRT-PCR and WB analyses. **(G)** MYC knockout attenuates LIN28B-driven proliferation in EC cells (CCK-8 assay). **(H, I)** Downregulation of CDK4 and 6 expression following MYC knockdown, validated by qRT-PCR and WB. **(J)** The clonogenic capacity of EC cells was conducted using clone formation assay. **(K)** EdU assay was performed to assess the proliferation of EC cells. **(L)** Evaluation of invasive capacity was conducted using Transwell assay. **(M)** Evaluation of migration ability was conducted using Transwell assay. The data are presented as the mean ± SD and assessed by conducting Paired t-test for A (*n*=8) and one-way ANOVA with *post-hoc* Bonferroni for B-M (*n*=3); *P < 0.05, **P < 0.01, and ***P < 0.001.

We have identified that LIN28B exerts pro-tumorigenic effects in EC. To investigate the underlying mechanism, we conducted focused studies on MYC, a critical oncogenic factor and a key downstream target of the LIN28B/let-7 axis. Prior evidence indicates that LIN28B-mediated suppression of let-7 upregulates MYC expression, thereby driving tumor progression in multiple malignancies (20, 21). Given this established mechanistic link and MYC’s central role in oncogenesis, we analyzed the correlation between LIN28B and MYC expression to elucidate their functional interplay in EC. TCGA analysis revealed a positive LIN28B-MYC mRNA correlation in EC (**Figure 4A**). Furthermore, IHC analysis of eight paired tumor and adjacent normal tissues revealed markedly elevated MYC expression in tumor tissues (**Figure 4B**). To validate bioinformatics results, we employed qRT-PCR and WB. As depicted in **Figures 4C, D**, LIN28B knockdown significantly reduced MYC expression in EC cells. To further investigate this relationship, we established a KLE cell line overexpressing LIN28B and silenced MYC. The efficiency of LIN28B overexpression and MYC knockdown were validated via qRT-PCR and WB assays (**Figures 4E, F**). Subsequent CCK-8 and EdU assays indicated that MYC depletion abolished the proliferative effects of LIN28B on EC cells (**Figures 4G, J**). Additionally, MYC depletion attenuated LIN28B-induced upregulation of CDK4 and CDK6 (**Figures 4H, I**). The results of clone formation results demonstrate that MYC deficiency significantly reduces the clonogenic capacity of EC cells mediated by LIN28B (**Figure 4K**). To further examine the effect of MYC on modulating the tumor-promoting activities of LIN28B in EC, we performed transwell assays. Our results demonstrate that MYC deficiency markedly suppresses the migratory and invasive phenotypes triggered by LIN28B overexpression (**Figures 4L, M**). In addition, we performed MYC overexpression in LIN28B-silenced EC cells and compared with control cells. The results demonstrated that MYC overexpression significantly increased the proliferative, invasive, and migratory capacities of LIN28B-depleted cells (**Supplementary Figure S1**). Consequently, our results suggest that MYC mediates LIN28B-driven oncogenicity.

### 3.5 LIN28B modulates the immune microenvironment in EC

**Figure 5:**
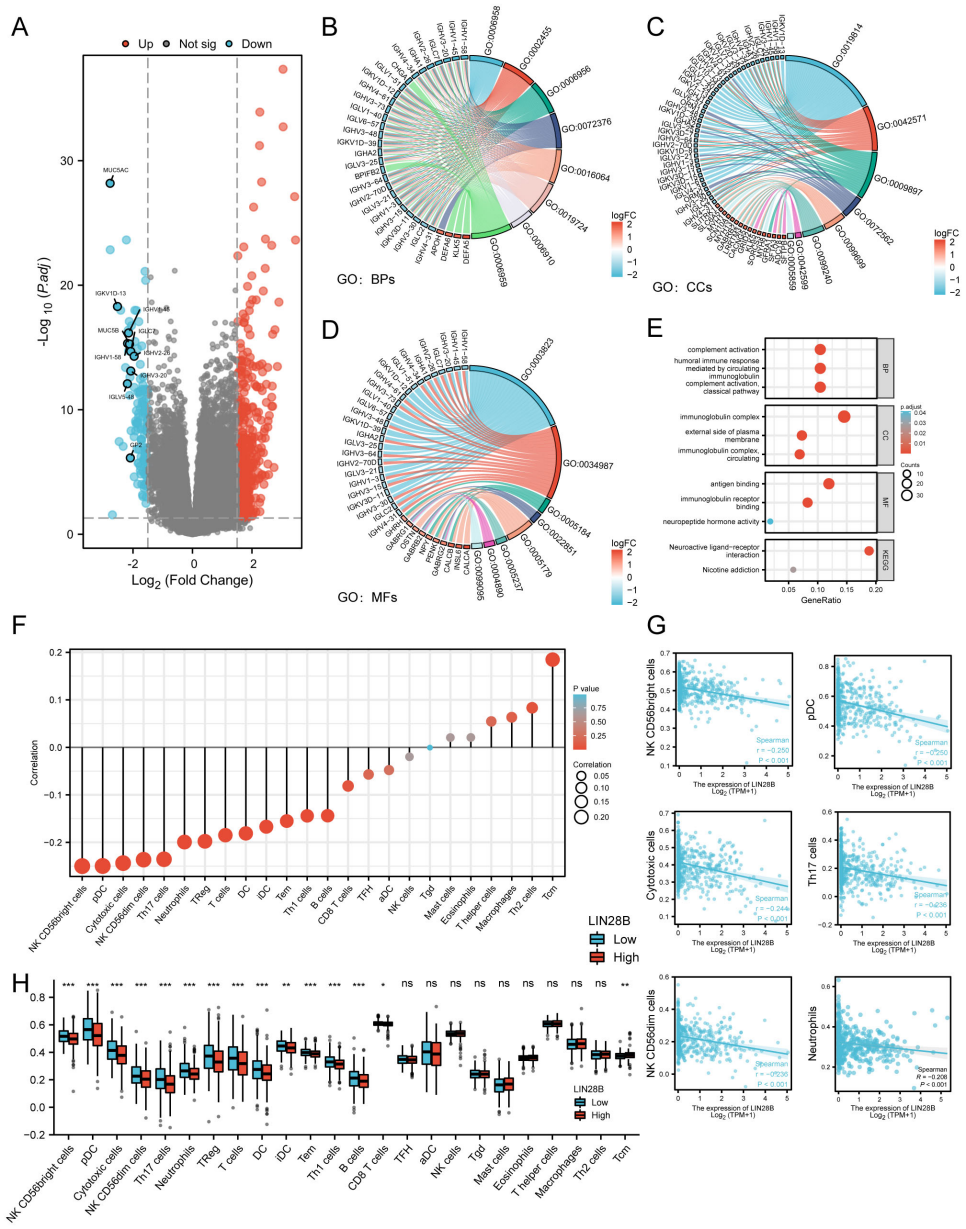
LIN28B regulates the immune microenvironment in EC. **(A)** Volcano plot displaying DEGs (blue: downregulated, red: upregulated). DEGs and immune-related genes from the ImmPort Database were intersected, identifying 50 downregulated immune genes, with the 10 most differentially expressed genes labeled in the volcano plot. **(B–D)** Chord plots visualizing top enriched GO terms for BPs, CCs, and MFs. **(E)** A bubble plot showcases significant enrichment of KEGG pathways and GO terms. Bubble sizes indicate the number of enriched genes. **(F)** A Lollipop Chart displays the correlation between LIN28B and immune cells, with dot size representing the degree of correlation. **(G)** Significant correlations between LIN28B expression and infiltration levels of NK CD56bright cells, pDCs, cytotoxic cells, NK CD56dim cells, Th17 cells, and Neutrophils in EC. **(H)** Differential immune cell enrichment scores between high-LIN28B (red) vs low-LIN28B (blue) groups.

LIN28B has been demonstrated to correlate with tumor immune escape, as well as the immune microenvironment (22, 23). In this study, we identified 608 DEGs (479 upregulated and 129 downregulated) in EC samples from the TCGA database, through a comparative analysis of groups exhibiting low versus high LIN28B expression levels (**Figure 5A**). Notably, 50 immune-related genes were co-downregulated in the LIN28B low-expression group, demonstrating a significant link between LIN28B and immune modulation. To elucidate the functional implications of these DEGs, we performed GO and KEGG analyses. The enriched GO terms encompassed humoral immune responses mediated by circulating immunoglobulin, immunoglobulin receptor binding, immunoglobulin complexes, complement activation via classical pathways, antigen binding, neuropeptide hormone activity, and circulating immunoglobulin complexes. These results show a strong correlation between elevated LIN28B expression and immune responses, as shown in **Figures 5B–E**. Additionally, we investigated the relationship between LIN28B expression levels and the infiltration of immune cells within EC. Those results show a negative correlation between high LIN28B expression and 13 types of immune cells. Furthermore, the relative enrichment scores for 14 types of immune cells were much lower in samples with elevated LIN28B expression, as opposed to those with diminished expression (**Figures 5F, H**). The correlation between representative immune cell infiltration levels and LIN28B expression levels is presented in **Figure 5G**. Taken together, our data indicates that LIN28B expression level significantly influences immune infiltration within the EC microenvironment.

### 3.6 LIN28B is a potential prognostic indicator for EC

**Figure 6:**
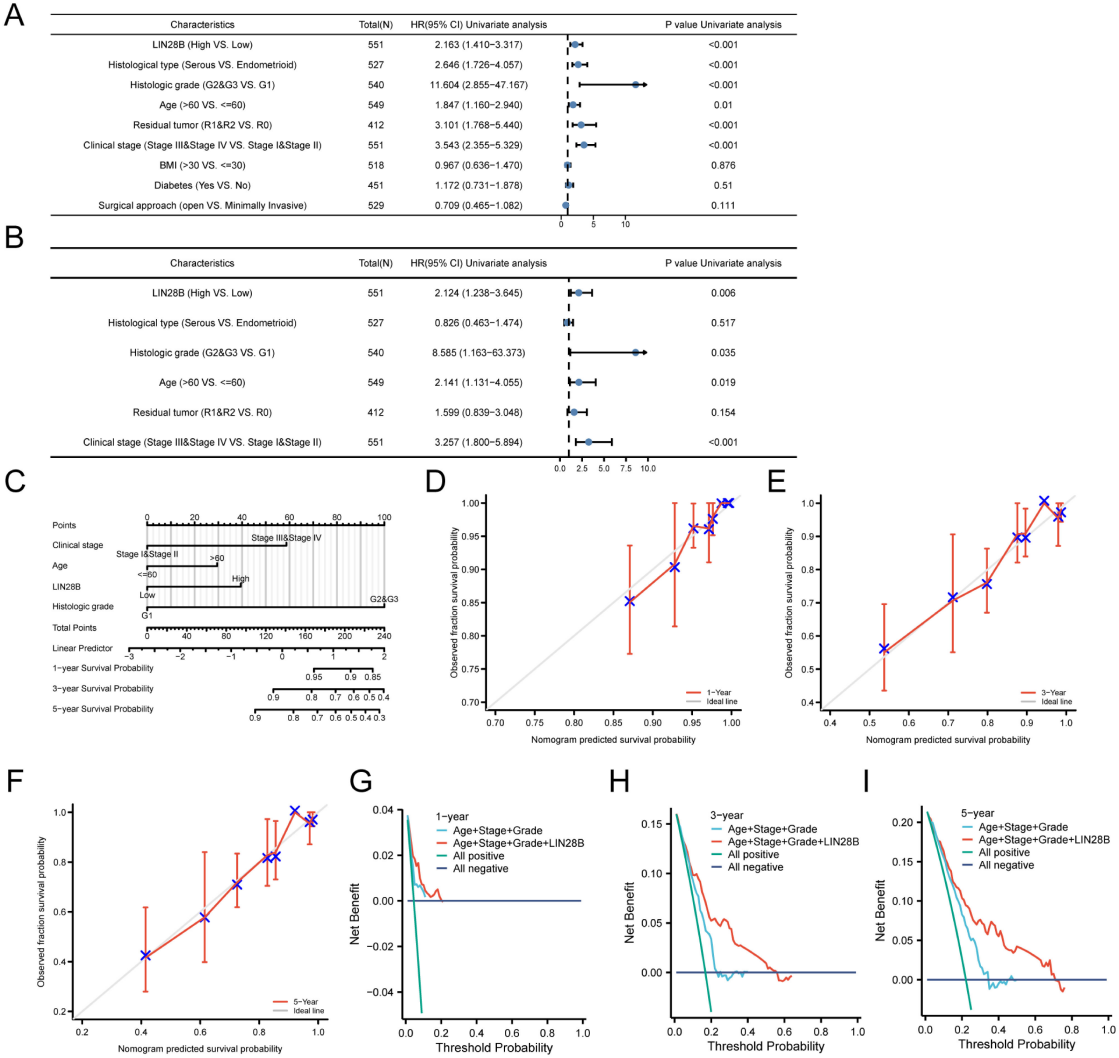
LIN28B is a potential prognostic indicator for EC. **(A)** A forest plot displays the outcomes of the univariate Cox analysis for LIN28B and additional clinical features. **(B)** A forest plot presents the multivariate Cox analysis, incorporating LIN28B expression, histological type, clinical stage, age, histological grade, and residual tumor. **(C)** A nomogram was constructed to predict the survival probabilities. **(D–F)** Calibration plots for the nomogram’s predicted survival probabilities are shown, with the gray line representing perfect prediction and the red line representing the prognostic model’s prediction. **(G–I)** DCA comparing nomogram models for 1-, 3-, and 5-year survival outcomes with (red) and without LIN28B expression (blue).

A univariate Cox regression analysis was employed to assess the predictive value of LIN28B expression, in conjunction with various clinicopathological factors, for predicting the OS of patients with EC. The forest plot revealed that several factors, including LIN28B expression, histological type, clinical stage, age, histological grade, and residual tumor, were significant predictors of survival in EC patients (**Figure 6A**). Furthermore, a multivariate Cox analysis identified LIN28B expression, clinical stage, age, and histological grade as independent prognostic indicators for OS in EC (**Figure 6B**). Based on those results (*P* < 0.05), we developed a prognostic nomogram. As depicted in **Figure 6C**, this prognostic model integrates LIN28B expression, age, clinical stage, and histological grade to predict patient outcomes, with a c-index of 0.760 (95% CI: 0.734–0.785). The mode employs a point system where each variable is assigned a specific number of points, ranging from 0 to 100. By summing the points for all variables, a total score is obtained, which corresponds to predicted 1-, 3-, and 5-year survival probabilities. The survival probabilities are ascertained through the intersection of a vertical line extending from the total points axis to the survival probability axis. As depicted in **Figures 6D–F**, the predicted survival probabilities align closely with the ideal prediction line, indicating a good model fit. The calibration curves for the predicted OS outcomes are consistent with the observed results. Furthermore, DCA was conducted to evaluate prognostic models with versus without LIN28B expression. The LIN28B-integrated model (red line) exhibited superior net benefit within the majority of clinical decision thresholds, confirming that LIN28B inclusion substantially improves prognostic accuracy and clinical utility (**Figures 6G–I**). Our results reveal the independent prognostic value of LIN28B expression in EC and suggest that this nomogram may serve as a reliable prognostic tool for EC patients.

## 4 Discussion

EC represents a growing global health challenge, with a notable rise in its global incidence from 1990 to 2019, now ranking as the fourth most common gynecologic malignancy in the United States (24, 25). Current evidence emphasizes that early risk stratification and tailored therapeutic interventions for high-risk subgroups substantially improve clinical outcomes (26). Consequently, investigating the mechanisms of EC tumor progression and identifying reliable high-risk factors is of utmost importance. Our study reveals that LIN28B drives EC progression through MYC-mediated oncogenesis while concurrently shaping an immunosuppressive tumor microenvironment. Consistent with prior findings, bioinformatics analysis revealed a significant LIN28B overexpression in EC tissues versus normal endometrium, correlating with a poor prognosis (8). Subsequently, functional validation through LIN28B knockdown in EC cell lines (HEC-1A, KLE) demonstrated significant reduction in proliferation, inhibition of migration, and suppression of invasion. Additionally, by generating an EC cell line with elevated LIN28B expression and MYC knockdown, we verified that MYC plays an important role in the oncogenic effects of LIN28B. Lastly, we demonstrated the correlation between LIN28B expression levels and the immune microenvironment of EC. A robust prognostic model based on LIN28B expression was developed and rigorously validated for its application in EC. These findings collectively establish LIN28B as both a therapeutic target and precision prognostic biomarker in EC.

LIN28B is widely recognized for its oncogenic properties, demonstrating aberrantly elevated expression levels across multiple tumor types (27). Mechanistically, LIN28B promotes tumorigenesis by driving malignant cell proliferation, facilitating invasive and migratory capacities, and establishing immunosuppressive microenvironments (11, 28, 29). Our bioinformatics analysis and IHC analysis revealed significant upregulation of LIN28B expression in EC tissues compared to normal controls. Additionally, KM survival analysis of the TCGA dataset indicated that EC patients with high LIN28B expression exhibited significantly poorer OS, PFI, and DSS. Experimental validation demonstrated constitutive LIN28B overexpression in EC cell lines, while siRNA-mediated LIN28B knockdown substantially attenuated cellular proliferation, invasion, and migration capacities. Collectively, our findings establish LIN28B as a crucial mediator of EC progression and a reliable predictor of adverse clinical outcomes.

The MYC family of proto-oncogenes, which consists of c-MYC (also referred to as MYC), N-MYC, and L-MYC, plays a crucial role in regulating cellular proliferation, metabolic programming, and apoptotic pathways (30). Notably, MYC emerges as one of the most pervasively activated oncogenic drivers across human malignancies (31). Functioning as a master transcriptional regulator, MYC affects tumor progression by regulating the expression of numerous genes, thereby governing critical biological cascades (32). Mechanistically, MYC’s oncogenic potency stems from its multidimensional regulation of proliferation, apoptosis, metabolic reprogramming, and immune microenvironment modulation, including immune evasion (33–35). Additionally, emerging evidence indicates that LIN28B promotes oncogenesis in gallbladder carcinoma, gastric carcinoma, and multiple myeloma via MYC transcriptional activation (20, 36, 37). The results of bioinformatics analysis identified a significant positive correlation between LIN28B and MYC mRNA expression in EC. WB and qRT-PCR results exhibited that MYC expression was dramatically downregulated following LIN28B knockdown. Additionally, rescue experiments further confirmed that MYC depletion markedly abrogated the pro-proliferative, invasive, and migratory phenotypes induced by LIN28B overexpression in EC cells. These results mechanistically establish LIN28B-mediated MYC upregulation as a crucial axis driving EC pathogenesis.

The tumor immune microenvironment plays a pivotal regulatory role in oncogenesis, exerting multifaceted influences on neoplastic proliferation, therapeutic resistance, metastatic dissemination, and immune evasion (38, 39). In EC, this microenvironment demonstrates dual clinical relevance, serving both as a driver of disease progression and a determinant of patient prognosis (40, 41). In this study, enrichment analysis results revealed that the biological functions of DEGs stratified by LIN28B expression levels revealed predominant enrichment in immunomodulatory pathways. Further bioinformatics analysis identified significant inverse correlations between LIN28B expression and tumor-infiltrating immune cell populations. Notably, elevated LIN28B expression inversely correlated with infiltration levels of 13 immune subtypes, including CD56^bright^ natural killer (NK) cells, plasmacytoid dendritic cells (pDCs), and neutrophils. CD56^bright^ NK cells suppress tumorigenesis in non-small cell lung cancer by targeting cancer stem cells and metastatic spread (42). Increased pDCs infiltration is associated with earlier tumor stages and improved survival (43), while cytotoxic neutrophils inhibit tumor progression and metastasis in breast cancer (44). These findings suggest that LIN28B serves as a potential immunomodulatory hub in EC, and therapeutic targeting of this molecule may represent a promising strategy to counteract tumor immune evasion.

Additionally, we developed a nomogram incorporating LIN28B expression with established clinicopathological parameters. The prognostic model demonstrated high predictive accuracy, evidenced by concordance between predicted and observed OS probabilities at 1-, 3-, and 5-year intervals through calibration curves. These findings position LIN28B as a promising prognostic biomarker for EC.

Notwithstanding these insights, certain limitations warrant consideration. First, while our findings suggest a potential link between LIN28B and tumor immunosuppression in EC, it is imperative to validate this mechanistic association through clinical studies correlating LIN28B expression levels with immune checkpoint markers and immune cell infiltration in patient tumor specimens. Second, while our database-derived prognostic model shows clinical potential, external validation using prospectively collected patient cohorts remains imperative. These critical gaps will be addressed in our subsequent research.

## 5 Conclusion

Our study confirms that LIN28B facilitates EC progression through the upregulation of MYC and mediates immunosuppressive tumor microenvironment reprogramming. Additionally, we establish LIN28B expression as an independent predictor of adverse clinical outcomes. These results suggest that LIN28B represents a therapeutic target and a robust prognostic indicator for EC.

## Data Availability

The original contributions presented in the study are included in the article/**Supplementary Material**. Further inquiries can be directed to the corresponding author.
