# Supplementary material for: LIN28B promotes the progression of endometrial cancer through upregulating MYC and correlates with immune microenvironment
Source: Front Oncol. 2025 Jul 16;15:1592193. doi: 10.3389/fonc.2025.1592193 (PMC12307211; doi:10.3389/fonc.2025.1592193)
Supplement: Supplementary file 1 [file DataSheet1.zip › Supplementary files/figure S1 legend.docx]

**Figure S1 MYC overexpression reverts tumor-suppressive effects induced by LIN28B silencing in EC**

(A) Validation of MYC overexpression and LIN28B knockdown efficiency through WB assays. (B) MYC overexpression promoted growth ability in EC cells (CCK-8 assay). (C-D) Upregulation of CDK4 and 6 expression following MYC overexpression, validated by qRT-PCR and WB. (E) The clonogenic capacity of EC cells was conducted using clone formation assay. (F) Evaluation of invasive capacity was conducted using Transwell assay. (F) Evaluation of migration ability was conducted using Transwell assay. (H) EdU assay was performed to assess the proliferation of EC cells. The data are presented as the mean ± SD and assessed by conducting unpaired t-test, *n* = 3 for each group; *P < 0.05, **P < 0.01, and ***P < 0.001
