# Supplementary material for: LIN28B promotes the progression of endometrial cancer through upregulating MYC and correlates with immune microenvironment
Source: Front Oncol. 2025 Jul 16;15:1592193. doi: 10.3389/fonc.2025.1592193 (PMC12307211; doi:10.3389/fonc.2025.1592193)
Supplement: Supplementary file 1 [file DataSheet1.zip › Supplementary files/Table S1.docx]

| Genes | Forward primer | Reverse primer |
| --- | --- | --- |
| *GAPDH* | GGTCTCCTCTGACTTCAACA | GTGAGGGTCTCTCTCTTCCT |
| *LIN28B* | AGCCCCTTGGATATTCCAGTC | AATGTGAATTCCACTGGTTCTCCT |
| *MYC* | GCAGCTGCTTAGACGCTGGATTTT | GCAGCAGCTCGAATTTCTTCCAGA |
| *CDK4* | GGAGGAGGAGGTGGAGGA | GTCCATCAGCCGGACAAC |
| *CDK6* | TGCACAGTGTCACGAACAGA | TTGATTTTGGAGGGATCTCG |

Table S1 The primer sequences
