# Supplementary material for: LIN28B promotes the progression of endometrial cancer through upregulating MYC and correlates with immune microenvironment
Source: Front Oncol. 2025 Jul 16;15:1592193. doi: 10.3389/fonc.2025.1592193 (PMC12307211; doi:10.3389/fonc.2025.1592193)
Supplement: Supplementary file 1 [file DataSheet1.zip › Supplementary files/Table S2.docx]

| Antibody | Dilution | Company | Product# |
| --- | --- | --- | --- |
| CDK4 | 1:1000 | Affinit | DF6102 |
| CDK6 | 1:1000 | Affinit | DF6448 |
| LIN28B | 1:1000 | abcam | ab191881 |
| MYC | 1:1000 | abcam | ab32072 |
| GAPDH | 1:1000 | Affinit | AF7021 |

Table S2 Antibodies
