# Supplementary figures and images for: LIN28B promotes the progression of endometrial cancer through upregulating MYC and correlates with immune microenvironment
Source: Front Oncol. 2025 Jul 16;15:1592193. doi: 10.3389/fonc.2025.1592193 (PMC12307211; doi:10.3389/fonc.2025.1592193)

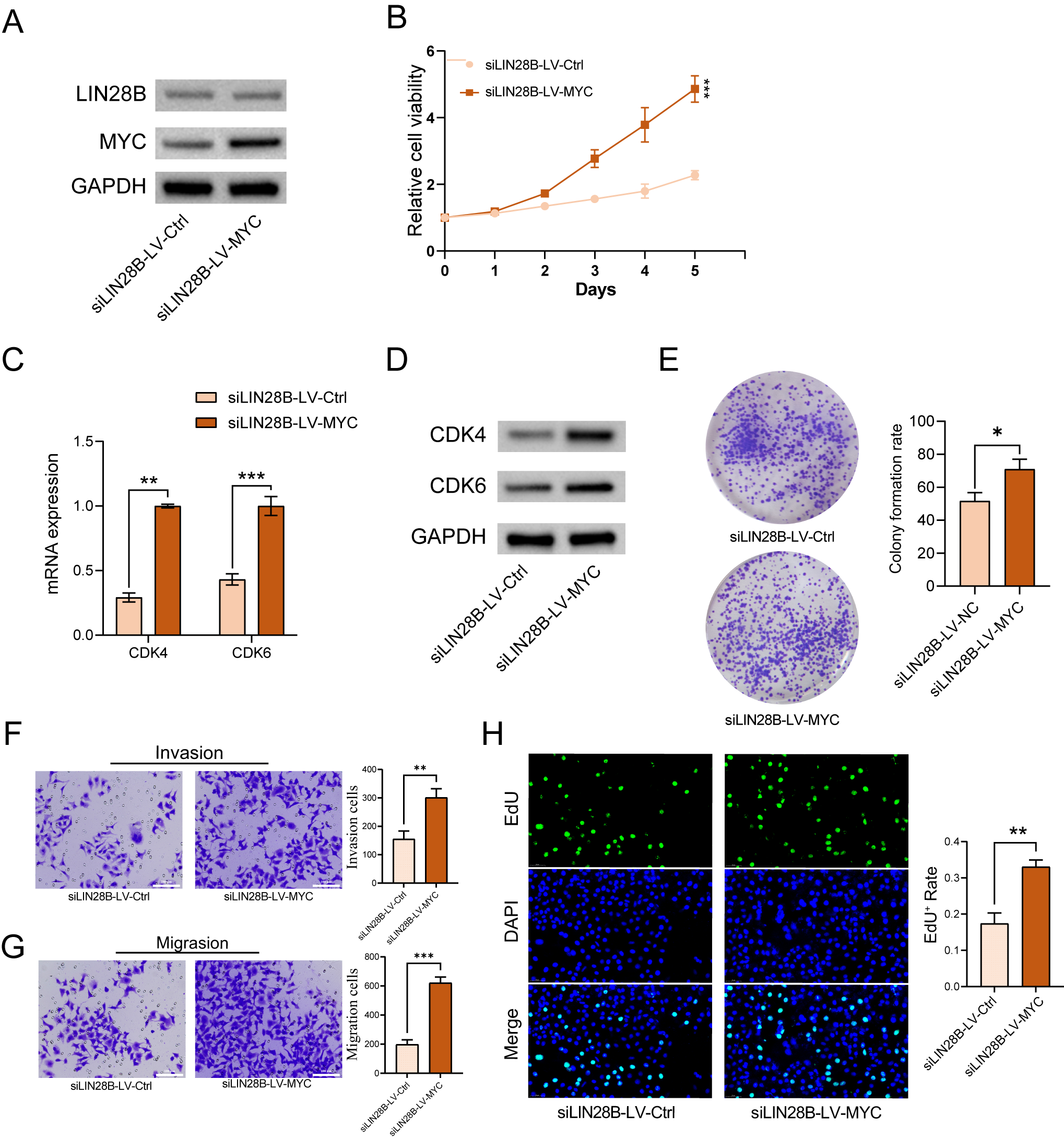

Supplement: Supplementary file 1 [file DataSheet1.zip › Supplementary files/FIG-S1.tif]
